# Deep-Learning-Based Morphological Feature Segmentation for Facial Skin Image Analysis

**DOI:** 10.3390/diagnostics13111894

**Published:** 2023-05-29

**Authors:** Huisu Yoon, Semin Kim, Jongha Lee, Sangwook Yoo

**Affiliations:** AI R&D Center, Lululab Inc., 318 Dosan-daero, Gangnam-gu, Seoul 06054, Republic of Korea

**Keywords:** facial skin feature segmentation, ground truth generation, facial wrinkles and pores, semantic segmentation, positional encoding, prior information, attention

## Abstract

Facial skin analysis has attracted considerable attention in the skin health domain. The results of facial skin analysis can be used to provide skin care and cosmetic recommendations in aesthetic dermatology. Because of the existence of several skin features, grouping similar features and processing them together can improve skin analysis. In this study, a deep-learning-based method of simultaneous segmentation of wrinkles and pores is proposed. Unlike color-based skin analysis, this method is based on the analysis of the morphological structures of the skin. Although multiclass segmentation is widely used in computer vision, this segmentation was first used in facial skin analysis. The architecture of the model is U-Net, which has an encoder–decoder structure. We added two types of attention schemes to the network to focus on important areas. Attention in deep learning refers to the process by which a neural network focuses on specific parts of its input to improve its performance. Second, a method to enhance the learning capability of positional information is added to the network based on the fact that the locations of wrinkles and pores are fixed. Finally, a novel ground truth generation scheme suitable for the resolution of each skin feature (wrinkle and pore) was proposed. The experimental results revealed that the proposed unified method achieved excellent localization of wrinkles and pores and outperformed both conventional image-processing-based approaches and one of the recent successful deep-learning-based approaches. The proposed method should be expanded to applications such as age estimation and the prediction of potential diseases.

## 1. Introduction

The face of a person has both universality, allowing recognition of a human face, and individuality, enabling distinction among individuals. These characteristics are used in applications such as face detection and face recognition. Because facial skin also exhibits these characteristics, facial features can be defined, classified, and analyzed. This information can detail the skin’s health status. Facial skin images can be captured using photographs for skin analysis or obtained by detecting the facial region in a person’s image and cropping it to extract the region of interest.

Representative skin features include wrinkles, pores, dark circles, and hyperpigmentation. The analysis of such skin features is relevant to dermatology or cosmetic recommendations for the beauty and anti-aging industries. Grouping skin features with similar characteristics and detecting them together could be more efficient. Specifically, wrinkles and pores can be grouped as morphological features, and dark circles, redness, and pigmentation can be grouped as colorimetric features. In this study, we focused on structural facial features, such as wrinkles and pores, and proposed a deep neural network for simultaneously segmenting both effectively.

Before the advent of deep learning, image processing techniques were used for wrinkle and pore detection. Automatic detection of wrinkles was widely performed using the Hessian or Gabor filter [1,2]. A Hessian matrix is defined as a square matrix of second-order partial derivatives. Eigen vectors and their corresponding eigen values represent the direction and magnitude at a specific position. The Gabor filter is a bio-inspired feature extraction filter consisting of Gaussian filtering and trigonometric modulation functions. Both filters extract features from a local area without considering a larger area. For facial pore segmentation, image-processing-based approaches such as high-pass filtering, k-means clustering, and morphological processing are effective [3]. In image processing approaches, the area of interest should be predefined for detecting pores and filter parameters should be optimized for specific images because the performance of the approaches is highly affected by skin and lighting conditions.

Research using deep learning has begun to emerge in the facial skin analysis field as well [4,5,6,7,8,9]. In [4], a deep learning model was used for segmenting acne, pigmentation, and wrinkles. A study [5] suggested a two-step wrinkle removal algorithm, where the first step is wrinkle segmentation. In both studies [4,5], U-Net++ was used as the segmentation model, which is advantageous for capturing more contextual information by incorporating multiple nested pathways. Furthermore, convolutional neural networks (CNNs) have been used to detect nasolabial folds, a type of wrinkle [6,7]. Deep learning is also being used in skin pore segmentation. For instance, in [8], U-Net with L1 loss was used for pore segmentation, and [9] suggested a shallow CNN focusing on pores with a simple shape.

In this study, we proposed a comprehensive deep learning approach for simultaneous segmentation of wrinkles and pores. To the best of the authors’ knowledge, this study is the first study focusing on detecting facial wrinkles and pores simultaneously and insights into the simultaneous detection of similar skin attributes using neural networks. Instead of focusing on proposing the new structural aspects of a deep neural network, we aimed to generate ground truth (GT) data that fit the characteristics of wrinkles and pores and propose a method that emphasizes the typical locations of each feature.

Specifically, the proposed method can be summarized by three features. First, based on the U-Net architecture, an attention mechanism was integrated to refine feature maps and enable their transmission to the decoding part. Second, a texture map with image processing filters that enhance the high-frequency details for wrinkles and pores was used to generate GT data. Finally, to improve the localization ability of the deep neural network, a novel model was designed to focus on major areas of wrinkle and pore occurrence, which are fixed in the facial region. This result was achieved using a computationally lightweight zero-padding technique, which improved simultaneous detection performance and reduced false positives.

This paper is organized as follows: Section 2 presents a review of studies related to the proposed method. In Section 3, we propose a facial-skin-specific segmentation network. Section 4 details the experimental results, compares them with existing image-processing-based methods, and verifies the effectiveness of the added techniques in the network. In the Discussion section, directions for future studies are detailed, and the paper is concluded.

## 2. Related Work

In this section, we briefly review the existing literature related to the proposed methodology.

### 2.1. U-Net and Its Variants

The U-Net is a U-shaped CNN that was originally devised for biomedical image segmentation [10]. The network consists of an encoder–decoder structure with skip connections that concatenate feature maps from the encoder to feature maps at the decoder to retain high-resolution information that can be lost during contracting paths in the encoding part. However, this simple skip connection can transfer noise from the encoder to the decoder, which can cause performance degradation.

To address the limitation of simple skip connections, studies have considered refining feature maps before skip connections. For example, a study [11] proposed using attention mechanisms to weight the feature maps from the encoder using the information from both the encoder and decoder before concatenating them with the decoder feature maps to help the network focus on the most relevant information and erase irrelevant information.

As another improvement strategy, dense skip connections have been used. For instance, dense U-Net is a modification of the U-Net architecture that incorporates dense skip connections between the encoder and decoder blocks, where feature maps from all preceding encoder blocks are concatenated with feature maps from the decoder [12]. In U-Net++ [13], the network was designed to learn complex interactions between feature maps across various levels of the encoder and decoder. These methods improve performance compared with U-Net, but they also increase model complexity.

### 2.2. Semi-Automatic Labeling for Ground Truth Generation

In a semantic image segmentation problem, each pixel is classified according to the predefined classes. The ImageNet dataset is widely used in computer vision [14], and the input images for this dataset typically have a size of 224×224. For images used in skin analysis, a considerably larger size is typically used, and pixel-level annotation accuracy is required, rendering elaborate labeling of skin features a challenging and time-consuming task in a supervised method.

To overcome this problem, a rough manual annotation concept was introduced [15] for wrinkle segmentation. With regard to the generation of the GT, manual labeling is performed with a thickness that sufficiently includes wrinkles first. Next, a wrinkle-enhancing image filter is applied to the input image. Finally, the filtered image and the annotation image are multiplied to obtain the GT with well-defined boundaries. In this study, we expanded this concept for general morphological feature segmentation in skin images.

### 2.3. Use of Positional Information

A research trend is to include positional information in the learning network to improve performance. First, positional information was used in transformers for natural language processing tasks [16], where the position of each element is encoded in sequence into input representation. Positional encoding has been extended to computer vision to develop the vision transformer (ViT) [17]. In the ViT, a self-attention mechanism is applied to image patches and positional embeddings have been used to encode spatial information, which allows the model to capture both local and global features of an image.

Moreover, the extension of positional encoding to CNNs can considerably improve image segmentation accuracy. In CNN-based segmentation schemes, positional encoding has been used to improve the accuracy of segmentation models. By incorporating positional encoding into CNNs, the model can handle the spatial relationship between pixels and generate accurate segmentation masks. CoordConv is an example of a method incorporating positional information into the CNN architecture by concatenating a coordinate channel that encodes the x- and y-coordinates of each pixel [18]. CoordConv enables the model to better learn the spatial relationship between pixels, which leads to more accurate segmentation results.

In CNN applications, positional encoding is extended beyond explicit usage at input, such as in the case of CoordConv. For instance, positional embeddings can be used not only in the input but also in the decoding part. The decoder was equipped with positional embeddings that were added to the output of the encoder, which allowed the decoder to improve disentangling and reconstruction accuracy [19]. Furthermore, there are analytical research results on whether CNNs learn implicitly positional information [20], where the authors mention the effect of zero-padding in the CNN.

## 3. Materials and Methods

### 3.1. Shape Prior-Driven Ground Truth Generation

**Figure 1:**
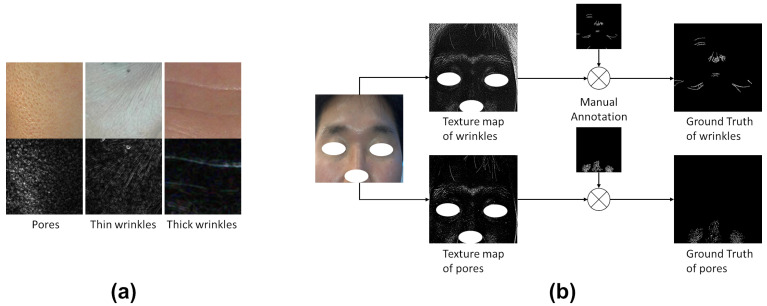
The first row of (**a**) shows examples of pores, thin wrinkles, and thick wrinkles, respectively. In the second row of (**a**), edge detection algorithms make their differences more clearly visible in grayscale. In (**b**), Ground Truth generation process is represented, where texture maps and manual annotations are multiplied to generate the GT.

In this subsection, we examined wrinkles and pores from the perspective of image signal processing. As displayed in Figure 1a, pores are clusters of small openings, whereas wrinkles can form straight and curved edges with varying thickness. This shape information can be integrated into the GT generation process. GT generation in this study is based on [15], where manual annotation was refined by multiplying with a texture map to yield the final GT, as displayed in Figure 1b. A texture map refers to an image that highlights the key areas of interest to be detected, such as a high-pass filtered image. Table 1 summarizes the formulas used to generate texture maps for wrinkles and pores, respectively. Detailed explanations for each formula are provided in the following:

**Table 1:** Formulation for texture map generation.

Skin Feature	Formulation	Eq
Wrinkle	$T\left(x,y\right)=\left(1-\frac{I(x,y)}{1+I_{G_{\sigma }}\left(x,y\right)}\right)\times 255$	(1)
Pore	$T\left(x,y\right)=t\cdot 1_{|t|>Th},$	(2)
	where $t=-\left(L_{0}\left(x,y\right)+\mathrm{Expand}\left(L_{1}\right)\left(x,y\right)\right)$	

#### 3.1.1. Ground Truth Generation for Wrinkles

For wrinkles, many well-known edge detection algorithms can be excellent candidates for creating the texture map. Unlike pores, wrinkles have connected lines, so the application of filters that emphasize coherence can be considered. In this study, we used the filter proposed by [15]. The filter is formulated using a Gaussian kernel with parameter σ as follows:

(1) $$T\left(x,y\right)=\left(1-\frac{I(x,y)}{1+I_{G_{\sigma }}\left(x,y\right)}\right)\times 255,$$

where *I* is the grayscale image and *T* is the resultant texture map. This image filter benefits from the smoothing effect on the surrounding area because of the Gaussian filter, which enhances the coherence between edges and renders it computationally simple and fast. After generating the texture map, the GT can be created by applying thresholding and multiplying it with the manual annotation map, just as in the case of pores.

#### 3.1.2. Ground Truth Generation for Pores

Pores are numerous small openings typically located on cheeks and nose. Each pore consists of a few pixels with a lower signal magnitude than their surroundings. Pore detection starts similarly to wrinkles, by converting the image into a grayscale format. To extract the high-frequency signal components of pores, the Laplacian pyramid technique is used. The formula for generating a texture map of pores is as follows:

(2) $$T\left(x,y\right)=t\cdot 1_{|t|>Th},\;\;\mathrm{where}\;t=-\left(L_{0}\left(x,y\right)+\mathrm{Expand}\left(L_{1}\right)\left(x,y\right)\right),$$

where $L_{i}$ represents Laplacian pyramid [21] at *i*-th level, and Expand denotes expanding operator consisting of upsampling and interpolation. The texture map was obtained after hard threshing with threshold Th, which clearly delineates the boundaries between the pores. The negative sign at the beginning of the formula is used to make pores, which typically have lower values compared with their surroundings in facial images, which is a positive sign in the texture map.

### 3.2. Implicit Learning of Occurrence Location

**Figure 2:**
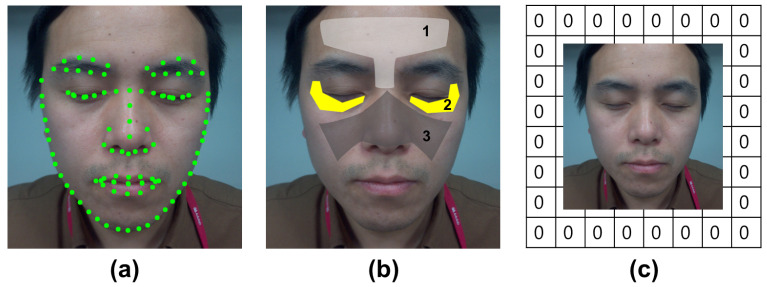
Human face has universality, which allows extraction of specific locations and analysis in those areas. (**a**) Example of facial landmark detection; (**b**) 1 (T zone): measurement areas of forehead and glabellar wrinkles; 2: measurement areas of eye wrinkles; 3 (butterfly zone): measurement areas of pores. (**c**) An example of a zero-padded input image.

Because the human face has universal physical characteristics, facial landmark detection can be performed by locating predefined landmarks on the face (Figure 2a). Specific areas of interest for analysis can be accessed through landmark detection. Facial landmarks have attracted considerable research attention because of their potential for application in many practical fields, such as face recognition [22].

Facial skin analysis can be based on the observations of universal phenomena that appear on the face. Generally, the locations where wrinkles and pores appear are predetermined, unlike acne or pigmentation, which can appear in various parts of the face. For example, wrinkles appear on the forehead, between the eyebrows (T zone), under the eyes, and beside the eyes (crow’s feet), whereas pores are distributed in the area of the nose and cheeks, also known as the butterfly zone. Their typical occurrence region is denoted in Figure 2b. Based on these observations, focusing on areas with a high probability of detecting each class can upgrade segmentation model performance.

To ensure the model focused on the areas of main occurrence, we enhanced the ability of the CNNs to learn location information. If the positional information is better learned, then it can not only improve segmentation performance in the regions of interest but also reduce false positives, that is, detection in incorrect locations. Therefore, we introduced zero-padding proposed in [20] to enhance the ability of CNN to learn location information implicitly and enable CNN to discriminate and learn the location of each item in the simultaneous detection of wrinkles and pores. Zero-padding is simple and does not increase computational complexity. Through the use of zero-padding, the network can better learn the expected locations and reduce detection errors at incorrect positions, which is demonstrated in detail in the Results section.

If zero-padding is the process at the input stage, attention mechanisms can improve the segmentation performance inside the neural network. The first attention mechanism was proposed in natural language processing to mimic human attention processes [23]. Attention allows the network to selectively focus on the target parts of an input while processing. In the proposed method, spatial attention and additive attention modules were proposed to emphasize processing at the target location. More details are discussed in the following subsection.

### 3.3. Network Architecture

**Figure 3:**
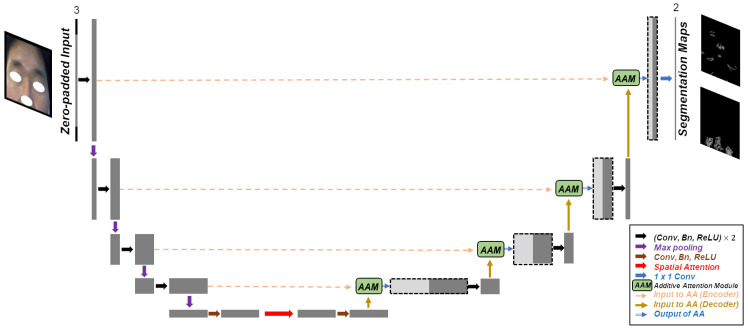
Architecture of the proposed network. The three-channel input is zero-padded and given to the network. The output consists of the two segmentation maps for wrinkles and pores, respectively.

Figure 3 displays the architecture of the proposed network. The network has a U-shaped framework, and most processing blocks apply twice the serial process of 3×3 convolution, batch normalization, and ReLU, as in other CNN-based networks. However, to make use of complex networks with better performance is not within the scope of this paper. This paper focused on learning location information within the face and produced discriminative results from wrinkle–pore simultaneous segmentation.

The proposed network has three main features. First, zero-padded input is used to facilitate the learning of positional information. The input to the network is zero-padded to have size (Z+H)×(Z+W)×C, where *Z* represents padding size. The output size is (Z+H)×(Z+W)×2, where 2 denotes that the segmentation maps of wrinkle and pore are yielded in concatenated form. The second feature is the application of spatial attention at bottlenecks. The attention scheme is applied to focus on the interest region. In spatial attention, max pooling and average pooling are performed on the feature map, yielding the two-channel output by concatenating two pooled features. Next, convolution and activation functions are used to yield a spatial attention map. Because attention was applied at the deepest layer, the refined effect of the feature map continued to be applied along the expanding path.

**Figure 4:**
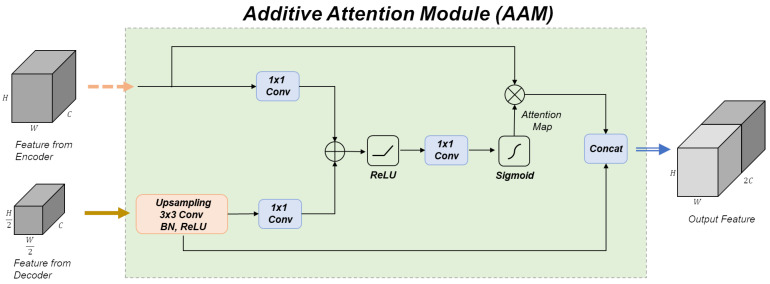
Schematic of the additive attention module. Input and output arrows follow the same convention as the network architecture in Figure 3.

The final feature is the suggestion of an additive attention module. Additive attention originally had the following form [24]:

(3) $$f\left(q,v\right)=v^{T}\sigma \left(W_{1}k+W_{2}q+b\right),$$

where q,k, and *v* represent query, key, and value, respectively. Here, σ denotes the activation function. In [11], additive attention was applied as the “attention gate”. Additive attention was used in this study, and we suggest the additive attention module (AAM), as in the green box in Figure 3 and described in detail in Figure 4.

As displayed in Figure 4, the feature maps from the decoding part serve as the query, while the feature maps from the encoder take the roles of key and value. The feature maps from the decoder go through upsampling, convolution, batch normalization, and ReLU activation in sequence. Next, an additive attention map is generated and multiplied with the feature maps from the encoder. Then, the two feature maps are concatenated and passed as input to the next block. By using these three techniques, a network was constructed to learn facial positions more accurately and emphasize important positions in a simple manner.

## 4. Results

### 4.1. Experimental Setup

For the dataset, we collected 314 facial images acquired from skin diagnosis devices, such as Lumini Kiosk V2 [25]. The images were cropped to include the forehead, eye, and butterfly zone, and then resized to 768×640. Among the cropped images, 264 images were selected for training, and the remaining 50 images were used for validation. Data labeling was performed by trained annotators. The input is a color image of size H×W×C, where *C* is the number of RGB channels. The output is a group of grayscale segmentation maps with size H×W×2, resulting in wrinkle and pore segmentation maps together in concatenated form. For training, MSE loss was selected to consider the pixel magnitude on GT. We trained our model using PyTorch with an NVIDIA GeForce RTX 3060.

### 4.2. Comparison with Existing Methods

**Figure 5:**
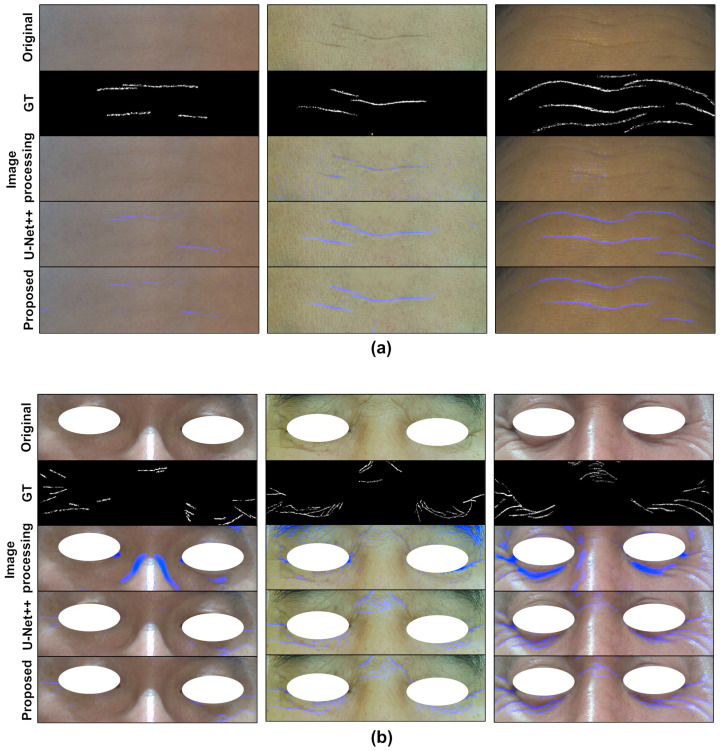
Comparison of wrinkle segmentation on (**a**) the forehead and (**b**) the eye regions. Each row represents the original image, GT, the result of the Frangi filter [26], the result of U-Net++, and the proposed network, respectively.

Figure 5 displays the comparison results of the representative image processing method, U-Net++, and the proposed method on wrinkle area. Each column represents a different subject. For image processing, a Hessian-based Frangi filter was selected [26]. Figure 5a displays the result on the forehead region. As displayed in the third row of the left column, the Frangi filter detects thick wrinkles well. However, if the lighting conditions are varied and wrinkles do not seem clear, the performance of the filter is poor. The results are not free from numerous false positives. By contrast, U-Net++ and the proposed method segment wrinkles well under various lighting and skin conditions, similar to the marking on GT. Figure 5b displays the result in the eye region. As presented in the second row of GTs, the thickness of wrinkles varies among the three data points. The data in the leftmost column predominantly demonstrate thin wrinkles, and the data in the middle column demonstrate numerous wrinkles of medium thickness. Finally, the rightmost has fewer wrinkles, but they are thicker in size. Image processing approaches reveal degraded performance, especially when wrinkles have various thicknesses in the image. Deep-learning-based approaches (U-Net++, proposed) exhibit consistent performance for both thin and thick wrinkles under various light and skin conditions.

**Figure 6:**
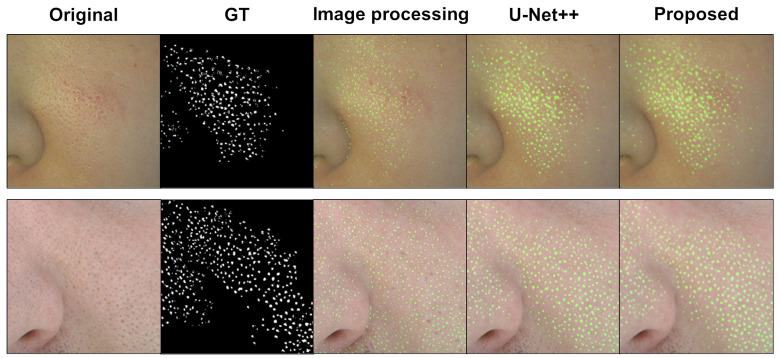
Comparison results of pore segmentation on the left cheek region. Each column represents the original image, GT, the result using the idea of [3], the result of U-Net++, and the result of the proposed network. Two rows indicate different subjects.

Figure 6 displays the results of pore detection on the left cheek area in the butterfly zone. The two rows represent two types of data. The third column of Figure 6 details the result of an image processing approach [3]. To implement the idea, high-frequency component extraction, k-means clustering, and morphological processing were applied one after the other. Therefore, most pores are detected well, but other high-frequency structures other than pores are also detected. The fourth and fifth columns detail the results of U-Net++ and the proposed network, respectively. Deep-learning-based methods demonstrate pore distribution that is similar to the GT.

Table 2 presents the intersection over union (IoU) values for wrinkles and pores, respectively. IoU is a measure that counts the overlap between the prediction and the GT. IoU is defined as follows:

(4) $$\mathrm{IoU}(X,\hat{X})=\frac{|X\cap \hat{X}|}{|X\cup \hat{X}|},$$

where *X* and $\hat{X}$ represent the GT and prediction, respectively. IoU values close to 1 denote superior prediction. For U-Net++ and the proposed algorithm, IoU values were calculated on the whole inference image. For image processing methods, the ROI of the target area was cropped in advance, and the IoU was calculated for selected images. As presented in Table 2, both deep-learning-based algorithms exhibited IoU values superior to image processing methods. In the comparison among deep learning algorithms, the proposed network exhibited slightly higher IoU values than U-Net++.

### 4.3. Model Evaluation

**Figure 7:**
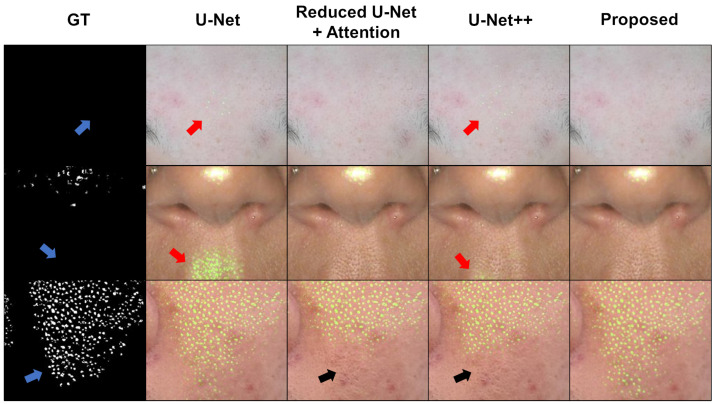
Compared results of deep neural networks for pore segmentation. Each row represents the different ROI of pore segmentation.

In Section 4.2, we compared the complete form of the proposed network with image-processing-based methods and a novel deep learning model, U-Net++. In this subsection, we investigate the effects of attention and zero-padding in the proposed network, which were introduced in Section 3.3. Figure 7 details the comparison results of the deep networks for pore segmentation. First, the result of the vanilla U-Net [27] was generated. The second model is a combination of the reduced U-Net and attention techniques. The reduced U-Net represents the small U-Net by halving the number of channels, resulting in a 1/4-sized model. Here, attention is termed to encompass both spatial attention and AAM. The fourth column displays the results of U-Net++. The proposed model in the fifth column is a combination of a downsized U-Net, attention modules, and zero-padding applied to the input.

The first row of Figure 7 reveals that the U-Net and U-Net++ detect the pores between eyebrows. Although they may appear as pores in a local area, a human annotator may not consider this during labeling because the area is in the T zone. No labels are visible in this area, as observed in the GT, as indicated by the blue arrow. By contrast, the reduced U-Net with attention and the proposed network do not detect these pores. The second row also exhibits a similar trend. The U-Net detects unwilling pores under the nose, as indicated by the red arrow. Although not as much as U-Net, U-Net++ also exhibits some degree of false positives. The remaining two models, the reduced U-Net with attention and the proposed one, do not detect false positives.

This observation reveals that attention mechanisms enable the model to focus on the areas that are most important, following the repetitive patterns of object locations in the GT images. However, with the reduction in false positives, a side effect of decreasing the detection area has been observed. In the third row of Figure 7, the area is indicated by a black arrow in the result of the reduced U-Net with attention. Even U-Net++ exhibits a similar reduction in the detection area. However, U-Net does not exhibit such a phenomenon. By contrast, the proposed method detected pores that were distributed very similarly to those in the GT without such a narrowing effect.

Table 3 presents the quantitative results of those models by ablation study. Two metrics, validation loss and IoU, are compared. A small loss indicates that the model is performing well in training and is making accurate predictions. The basic U-Net has the largest model size but the poorest performance among the evaluated models. The reduced U-Net with attention exhibited similar losses to the basic U-Net but considerably higher IoU values. Finally, the proposed model exhibited a significant improvement in both loss and IoU values, even with fewer parameters than U-Net. These qualitative and quantitative comparisons confirmed that the proposed network exhibited superior performance with a smaller capacity. These results alleviate concerns regarding the simultaneous detection of various features.

## 5. Discussion

The proposed network involved training the model to learn that wrinkles and pores are concentrated in certain facial zones, such as the T zones or butterfly zones, which are well-known areas for their appearance, and to use this information to improve detection accuracy. Examples of other research that considers the locations where the segmentation target is detected can be found in computer vision for autonomous driving purposes [28]. In [28], for multiclass segmentation environments for autonomous driving vehicles, certain classes have a defined range of locations they occupy. For instance, because cars cannot fly in the sky, they are predominantly distributed below a certain height in the image. To achieve this result, height-driven attention methods (HANET) have been proposed and have shown improved performance.

HANET and the proposed method utilize the location information of the segmentation target to improve model performance. However, HANET focuses on assigning higher weights to the locations where the segmentation target appears frequently. We enhanced CNN’s ability to learn location information. We introduced the zero-padding proposed in [20] to enhance the ability of CNN to learn location information implicitly and enable the CNN to better discriminate and learn the location of each item in the simultaneous detection of wrinkles and pores. Zero-padding is simple and does not increase computational complexity. Through the use of zero-padding, the network can better learn the expected locations and reduce detection errors at incorrect positions, as demonstrated in Section 4.3.

As mentioned in the introduction, skin attribute segmentation using deep learning techniques has attracted considerable research attention [4,5,6,7,8,9]. Although considerable advancements have been achieved in the application of state-of-the-art networks and their integration with a generative model to expand the scope of application, the unique characteristics of skin are yet to be incorporated into the network itself. The proposed study stands out from other approaches by considering the characteristics of skin in terms of generating GT based on image processing, designing the model to better learn the occurrence locations of skin features, and creating a compact model based on reduced U-Net. These methods incorporate the unique aspects of skin while leveraging state-of-the-art networks, which provides distinctiveness compared with other methods that simply adopt the latest networks.

The segmentation results of various skin features distributed on the face can be used to analyze skin characteristics. For example, severity scores can be assigned based on the area occupied by detected wrinkles or pores in a designated ROI. Alternatively, a score network can be connected to the segmentation network that starts from the deepest feature map of the segmentation network to directly train severity scores. These scores can be used for further analysis of skin characteristics. Furthermore, segmented results can also be used to calculate abstract measures, such as surface skin age, skin elasticity, and roughness. In the future, the segmentation results can be extended to automated skin analysis.

As a final point, we discuss the limitations of our proposed method and future directions for improvement. First, although the proposed method was designed for the simultaneous segmentation of wrinkles and pores, we did not analyze the severity of the detected results. In our subsequent research, we will aim to propose an end-to-end integrated model of segmentation and analysis. Second, GT can be improved. The current semi-automatic labeling approach generates GT with somewhat uneven boundaries. Therefore, creating a more coherent GT using additional post-processing filters for wrinkles can improve performance. For pores, prior information, such as round shape, can be considered in creating a GT. Finally, we believe that it is necessary to consider an improved loss function. In this proposed method, we used L2 loss (MSE) to consider the magnitude information of the texture map, but we will investigate improved loss functions, such as Dice loss, which considers the imbalance of the GT, or a weighted loss function, which assigns weight to the loss of wrinkles and pores separately, in the future.

## 6. Conclusions

In this study, the first deep neural network for simultaneous segmentation of facial wrinkles and pores was proposed. The proposed model incorporates zero-padding and attention mechanisms to better learn positional information, and a novel ground truth generation scheme suitable for each skin feature was devised. The experimental results revealed that the proposed network outperforms existing methods, and the reinforcement of positional information in the network was confirmed to enhance the network’s ability to better learn the location of features within the face. In the future, we plan to extend the improved segmentation performance to automatic skin analysis, such as skin elasticity and roughness, using the proposed method.

## Figures and Tables

**Table 2:** Comparison of IOU values.

	Image Processing	U-Net++	Proposed
**IoU of Wrinkles**	0.0833	0.2160	0.2341
**IoU of Pores**	0.2886	0.3669	0.4032

**Table 3:** Ablation study.

	#Params	Loss	IoU of Wrinkle	IoU of Pore
U-Net	17.3 M	1.243	0.2078	0.3601
Reduced U-Net	4.3 M	1.250	0.2147	0.3646
Reduced U-Net, Attentions	5.2 M	1.242	0.2250	0.3714
Reduced U-Net, Attentions, Zero-padding (Proposed)	5.2 M	**1.145**	**0.2341**	**0.4032**

## Data Availability

Data sharing not applicable.
